# Particulate Air Pollution, Exceptional Aging, and Rates of Centenarians: A Nationwide Analysis of the United States, 1980–2010

**DOI:** 10.1289/EHP197

**Published:** 2016-05-03

**Authors:** Andrea A. Baccarelli, Nick Hales, Richard T. Burnett, Michael Jerrett, Carter Mix, Douglas W. Dockery, C. Arden Pope

**Affiliations:** 1 Department of Environmental Health, Harvard T.H. Chan School of Public Health, Boston, Massachusetts, USA; 2 Department of Economics, Brigham Young University, Provo, Utah, USA; 3 Environmental Health Directorate, Health Canada, Ottawa, Ontario, Canada; 4 Division of Environmental Health Sciences, School of Public Health, University of California Berkeley, Berkeley, California, USA

## Abstract

**Background:**

Exceptional aging, defined as reaching age 85 years, shows geographic inequalities that may depend on local environmental conditions. Links between particulate pollution—a well-recognized environmental risk factor—and exceptional aging have not been investigated.

**Objectives:**

We conducted a nationwide analysis of ~28 million adults in 3,034 United States counties to determine whether local PM2.5 levels (particulate matter < 2.5 μm in aerodynamic diameter) affected the probability of becoming 85- to 94-year-olds or centenarians (100- to 104-year-olds) in 2010 for individuals who were 55–64 or 70–74 years old, respectively, in 1980.

**Methods:**

We used population-weighted regression models including county-level PM2.5 from hybrid land-use regression and geostatistical interpolation, smoking, obesity, sociodemographic, and age-specific migration variables.

**Results:**

On average, 2,295 and 71.4 per 10,000 of the 55- to 64- and 70- to 74-year-olds in 1980, respectively, remained in the 85- to 94- and 100- to 104-year-old population in 2010. An interquartile range (4.19 μg/m3) increase in PM2.5 was associated with 93.7 fewer 85- to 94-year-olds (p < 0.001) and 3.5 fewer centenarians (p < 0.05). These associations were nearly linear, were stable to model specification, and were detectable below the annual PM2.5 national standard. Exceptional aging was strongly associated with smoking, with an interquartile range (4.77%) increase in population who smoked associated with 181.9 fewer 85- to 94-year-olds (p < 0.001) and 6.4 fewer centenarians (p < 0.001). Exceptional aging was also associated with obesity rates and median income.

**Conclusions:**

Communities with the most exceptional aging have low ambient air pollution and low rates of smoking, poverty, and obesity. Improvements in these determinants may contribute to increasing exceptional aging.

**Citation:**

Baccarelli AA, Hales N, Burnett RT, Jerrett M, Mix C, Dockery DW, Pope CA III. 2016. Particulate air pollution, exceptional aging, and rates of centenarians: a nationwide analysis of the United States, 1980–2010. Environ Health Perspect 124:1744–1750; http://dx.doi.org/10.1289/EHP197

## Introduction

Recent declines in mortality have resulted in a worldwide increase in exceptional aging, defined as reaching age 85 years or older [National Institute on Aging (NIA) 2013]. Persons ≥ 85 years of age comprise the fastest-growing segment of the world’s population. Between 2010 and 2050, the number of individuals ≥ 85 years old will increase worldwide by > 300%, and the number of centenarians is projected to rise as much as 10 times [NIA/World Health Organization (WHO) 2011]. The distribution of exceptional aging shows substantial geographic inequalities that may depend, at least in part, on local conditions (NIA/WHO 2011). Many cohort studies have shown that long-term exposure to air pollution, particularly to particulate matter < 2.5 μm in aerodynamic diameter (PM_2.5_), is associated with cardiovascular morbidity—which affects considerably aging individuals (Brook et al. 2010)—and increased total mortality (Beelen et al. 2014; Cesaroni et al. 2013; Crouse et al. 2012; Dockery et al. 1993; Jerrett et al. 2013; Miller et al. 2007; Pope et al. 2002; Zeger et al. 2008).

Although associations of PM_2.5_ exposures have been well documented, it is unclear how these associations extend to the extremes of the age distribution. Aging and mortality are certainly linked; however, simulation studies have shown key differences in their biological and statistical dynamics, particularly at the extreme end of the human life span (Olshansky et al. 2001). To date, no study has investigated whether exposure to air pollution adversely affects the probability of exceptional aging. For this analysis, we asked the following question: Using U.S. county-level data, is there evidence that PM_2.5_ air pollution affects population-based measures of exceptional aging? We hypothesized that counties with less air pollution, as well as those with favorable sociodemographic conditions, have higher probabilities of exceptional aging, even when controlling for other population-level health-influencing factors. We analyzed data on ~28 million individuals ≥ 55 years old across 3,034 counties to examine the association of PM_2.5_ concentrations and other potential determinants with the probability of aging to 85–94 years old and separately of becoming a centenarian (100–104 years old) in 2010, given the population in 1980.

## Methods

### Demographic and Socioeconomic Data

County-level demographic and socioeconomic data were drawn from the 1980, 2000, and 2010 censuses (U.S. Census Bureau 1980, 2000, 2010). Data from Hawaii and Alaska were excluded because of inadequate PM_2.5_ exposure estimates. The following county-level data for other demographic and socioeconomic variables were compiled from year 2000 census data: median age, percent of population > 65 years old, percent of black or Hispanic population, population density, percent of population in urban areas, median income, percent of high school graduates, percent below poverty level, and percent unemployed. Because of the importance of adequately addressing migration rates for the elderly, relevant age-specific migration rates were obtained for the decades of the 1980s, 1990s, and 2000s (Winkler et al. 2013). See Table 1 for a description and summary of these variables and their sources.

**Table 1:** Description of United States county-level variables with unweighted means (SD), interquartile ranges and sources.

Variable	Description	Mean (SD)	IQR	Source
$P_{85–94}^{\mathrm{EA}}$	(Number of people 85–94 years old in 2010 divided by number of people 55–64 years old in 1980) × 10,000	2,295 (779.5)	728.52	U.S. Census Bureau 2010 U.S. Census Bureau 1980
$P_{100–104}^{\mathrm{EA}}$	(Number of people 100–104 years old in 2010 divided by number of people 70–74 years old in 1980) × 10,000	71.4 (55.3)	56.30	U.S. Census Bureau 2010 U.S. Census Bureau 1980
PM_2.5_	Mean PM_2.5_ concentrations from 1999–2008 (μg/m^3^)	10.4 (2.8)	4.19	Beckerman et al. 2013
Percent smoking	Percentage of adults in county that smoked daily in 2000	21.5 (3.7)	4.77	Institute for Health Metrics and Evaluation 2014
Obesity prevalence	Average, age-adjusted percentage of population that was obese (data from 2004–2010)	28.1 (3.6)	3.53	CDC 2013
Median income	Median income in 1999 (in thousands of U.S. dollars)	35.3 (8.8)	9.60	U.S. Census Bureau 2000
Percent below poverty	Percentage of population below poverty line	13.3 (5.6)	6.90	U.S. Census Bureau 2000
Percent black	Percentage of population that was black in 2000	8.9 (14.6)	10.04	U.S. Census Bureau 2000
Percent Hispanic	Percentage of population that was Hispanic in 2000	6.2 (12.1)	4.19	U.S. Census Bureau 2000
Population density	Thousands of people per square mile in 2000	0.25 (1.67)	0.09	U.S. Census Bureau 2000
Percent urban	Percentage of population that live in urban areas in 2000	40.2 (30.9)	53.69	U.S. Census Bureau 2000
Percent high school graduate	Percentage of population that were high school graduates	77.3 (8.7)	12.70	U.S. Census Bureau 2000
Percent unemployed	Percentage of population that was unemployed	5.8 (2.7)	2.90	U.S. Census Bureau 2000
Population in 2000	Number of people living in a county in 2000	89,927 (293,515)	50,742	U.S. Census Bureau 2000
Median age	Median age for county in 2000 (years)	37.4 (4.0)	4.60	U.S. Census Bureau 2000
Percent < 65 years old	Percentage of population > 65 years old in 2000	14.8 (4.1)	4.97	U.S. Census Bureau 2000
Migration 1980, 55- to 60-year-olds	Migration rate for 55- to 60-year-olds in 1980s^*a*^	4.7 (18.9)	12.00	Winkler et al. 2013
Migration 1980, 60- to 64-year-olds	Migration rate for 60- to 64-year-olds in 1980s^*a*^	7.4 (22.3)	14.00	Winkler et al. 2013
Migration 1980, 70- to 74-year-olds	Migration rate for 70- to 74-year-olds in 1980s^*a*^	1.9 (13.5)	10.00	Winkler et al. 2013
Migration 1980, ≥ 75-year-olds	Migration rate for ≥ 75-year-olds in 1980s^*a*^	–0.43 (10.2)	10.00	Winkler et al. 2013
Migration 1990, 65- to 70-year-olds	Migration rate for 65- to 70-year-olds in 1990s^*a*^	10.1 (20.5)	17.00	Winkler et al. 2013
Migration 1990, 70- to 74-year-olds	Migration rate for 70- to 74-year-olds in 1990s^*a*^	4.2 (13.3)	12.00	Winkler et al. 2013
Migration 1990, ≥ 75-year-olds	Migration rate for ≥ 75-year-olds in 1990s^*a*^	–0.17 (14.0)	13.00	Winkler et al. 2013
Migration 2000, ≥ 75-year-olds	Migration rate for ≥ 75-year-olds in 2000s^*a*^	–0.81 (17.2)	15.00	Winkler et al. 2013
Abbreviations: IQR, interquartile range; PM_2.5_, particulate matter with aerodynamic diameter < 2.5 μm; SD, standard deviation. ^***a***^Age-specific migration rates calculated as (net migration over the given decade divided by expected population at the end of the decade) times 100, where net migration is observed final population minus expected final population.				

### Construction of Population-based Probabilities of Exceptional Aging

We constructed two age-specific, population-based indices of county-level probabilities of exceptional aging. The first index was the proportion of 85- to 94-year-old persons per 10,000 in the 2010 census relative to the population of 55- to 64-year-olds in the 1980 census, constructed as follows:


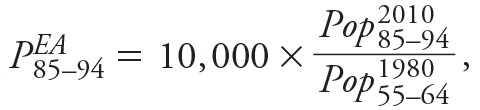
[1]

where *P^^EA^^*_85–94_ is the constructed proportion of exceptionally aged individuals 85–94 years old in 2010, *Pop* ^^2010^^_85–94_ is the population of 85- to 94-year-olds in 2010, and *Pop* ^^1980^^_55–64_ is the population of 55- to 64-year-olds in 1980. The second index was the proportion of 100- to 104-year-old persons per 10,000 in the 2010 census relative to the population of 70- to 74-year-olds in the 1980 census, constructed as follows:


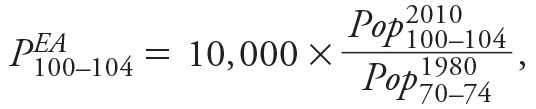
[2]

where *P^^EA^^*_100–104_ is the constructed proportion of exceptionally aged individuals 100–104 years old in 2010, *Pop* ^^2010^^_100–104_ is the population of 100- to 104-year-olds in 2010, and *Pop* ^^1980^^_70–74_ is the population of 70- to 74-year-olds in 1980. In the absence of migration, the indices above are simply scaled (× 10,000) probability ratios. With control for migration in statistical models, these indices are approximately equivalent to scaled probabilities of survival over the 30-year span for the respective age range. These variables are also summarized in Table 1.

### Air Pollution, Smoking, and Obesity Data

County-level exposures to PM_2.5_ were estimated using a hybrid approach that included land-use regression, traffic indicators, and Bayesian maximum entropy interpolation of land-use regression space-time residuals as documented elsewhere (Beckerman et al. 2013). The model is highly predictive of ground-level concentrations with a cross-validation *R* ^2^ of 0.79 and no indication of bias. These estimates were population-weighted averages (using the 2000 census data) from census-tract estimates averaged for all months of 1999–2008. The percentage of adults who smoked daily in 2000 was obtained for each county from the Institute for Health Metrics and Evaluation (2014), and obesity prevalence data were obtained from the Centers for Disease Control and Prevention (CDC 2013).

### Statistical Analysis

We used population-weighted regression, weighting by the square root of the total population in the year 2000, to estimate associations of probabilities of exceptional aging (*P^^EA^^*_85–94_ and *P^^EA^^*_100–104_) with PM_2.5_ levels and with other determinants. Primary results were obtained from a linear regression model including PM_2.5_ as an independent variable; smoking, obesity, socioeconomic, and demographic variables; indicator variables for the nine geographic census divisions of the United States; and age-specific migration rate variables. The age-specific migration rates included in the regression models were selected to provide the closest possible temporal alignment consistent with the initial age groups and with the relevant subsequent age groups for the entire three-decade study period. For *P^^EA^^*_85–94_ (persons who were 55–64 years old in the 1980 census), the most relevant and best temporally matched available migration rate data included the migration rates for ages 55–60 and 60–64 in the 1980s, the migration rates for ages 65–70 and 70–74 in the 1990s, and the migration rate for ages ≥ 75 in the 2000s. For *P^^EA^^*_100–104_ (persons who were 70–74 years old in the 1980 census), the most relevant and best temporally matched available migration rate data included the migration rates for ages 70–74 and ≥ 75 in the 1980s and the migration rates for ages ≥ 75 in the 1990s and the 2000s.

Sensitivity analyses employing various alternative regression models were conducted to evaluate the robustness of the study findings. The largest modeling concern involved adequately controlling for migration and accounting for outlier counties with extreme migration patterns. Therefore, models were estimated with and without migration variables and with and without census-division indicator variables. Models were also estimated using data from all counties, excluding observations with model residuals > 3 standard deviations from zero (censoring 37 observations) and excluding the 5% of counties (censoring ~150 observations) with the most extreme migration patterns (based on migration rates for ages ≥ 75 in the 2000s). In addition, because the precision of the exceptional aging variables was dependent upon county population, the regression models were weighted by the square root of the population. All linear models were estimated using the PROC REG procedure in SAS v.9.3 (SAS Institute Inc.). We also fit generalized additive models (GAMs) with a linear fit for PM_2.5_ and penalized regression smoothers (allowing for ≤ 4 degrees of freedom for each smooth) for the other covariates (excepting the census-division indicators). Finally, we also fit nonlinear models using a penalized regression smoother for PM_2.5_ in addition to the other covariates. These GAMs were estimated using the gam function in the R software mgcv package (R Core Team 2015).

## Results

### Probabilities of Exceptional Aging for Individuals 55–64 and 70–74 years old in 1980

In 1980, the U.S. population of the 48 contiguous states included 21,595,507 individuals who were 55–64 years old and 6,418,352 individuals who were 70–74 years old. In 2010, the 85- to 94-year-old group included 5,035,379 individuals and was therefore more than four times smaller than the corresponding group of 55- to 64-year-olds in 1980 (mean *P^^EA^^*_85–94_ = 2,295 individuals in 2010, scaled to 10,000 individuals in 1980). In 2010, the 100- to 104-year-old group included 48,303 individuals and was < 0.8% of the corresponding age group of 70- to 74-year-olds in 1980 (mean *P^^EA^^*_100–104_ = 71.4 individuals in 2010, scaled to 10,000 individuals in 1980).

### PM_2.5_ Levels and Probabilities of Exceptional Aging

**Figure 1:**
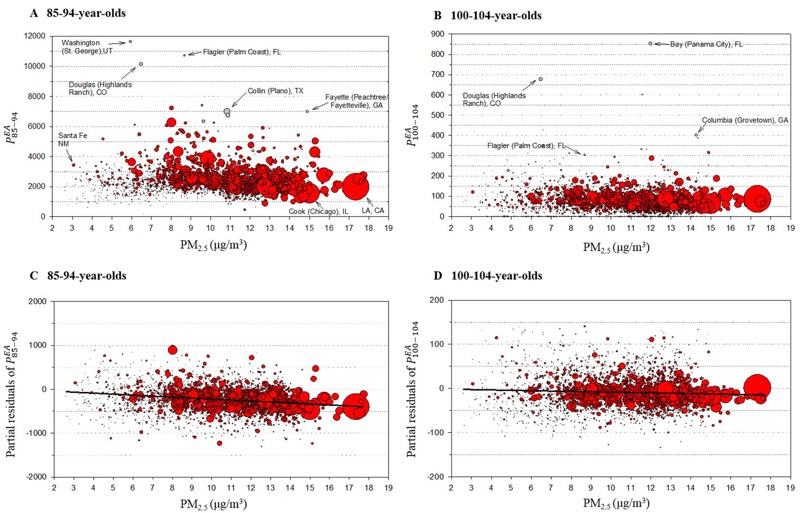
County-level particulate air pollution (PM_2.5_) and indices of exceptional aging, *P^EA^*_85–94 _(*A*) and *P^EA^*_100–104 _(*B*). Gray circles represent outlier counties. (*C*) and (*D*) present partial residuals obtained by excluding outlier counties and adjusting for all covariates except PM_2.5_ for both age groups, respectively. Bubble size represents the square root of the population in 2000. *P^EA^*_85–94_, (number of people 85–94 years old in 2010 divided by number of people 70–74 years old in 1980) × 10,000; *P^EA^*_100–104_, (number of people 100–104 years old in 2010 divided by number of people 70–74 years old in 1980) × 10,000; PM_2.5_, particulate matter with aerodynamic diameter < 2.5 μm.

Counties with higher PM_2.5_ concentrations had reduced probabilities of exceptional aging between 1980 and 2010. Plots of unadjusted county-level probabilities of exceptional aging for the two age groups (*P^^EA^^*_85–94_ and *P^^EA^^*_100–104_) showed negative correlations with PM_2.5_ levels but revealed the presence of several outlier counties (Figure 1A,B). We excluded these outliers and used regression models controlling for possible confounders, which were characterized through relevant variables for smoking, obesity, socioeconomic and demographic characteristics, age-specific migration rates, and regional indicators (Table 2). Plots of the corresponding partial residuals (residuals obtained by adjusting for all covariates except PM_2.5_) showed a subtle, near-linear covariate-adjusted association between PM_2.5_ and probabilities of exceptional aging (Figure 1C,D). The plots illustrate that unexplained variability in exceptional aging remained, particularly for the older group, but that the results were not driven by outliers.

**Table 2:** Regression coefficients (SE) for measures of exceptional aging regressed on PM_2.5_ and other covariates using the full linear models with censoring of observations with residuals > 3 standard deviations. Coefficients are scaled per interquartile range difference of each variable.

Variable (× IQR)	Difference in the rates of 85- to 94-year-olds^*a*^ ($P_{85–94}^{\mathrm{EA}}$)	Difference in the rates of 100- to 104-year-olds^*a*^ ($P_{100–104}^{\mathrm{EA}}$)
PM_2.5_ (× 4.19 μg/m^3^)	–93.7 (12.2)***	–3.5 (1.5)*
Percent smoking (× 4.77)	–181.9 (14)***	–6.4 (1.7)***
Percent obesity (× 3.53)	–83.9 (9.5)***	–3.1 (1.1)**
Median income (× 9.60)	62.5 (12.4)***	5.3 (1.5)***
Percent below poverty (× 6.90)	–60.5 (19.5)**	0.5 (2.4)
Population density (× 0.09)	0.2 (0.1)	0 (0)**
Percent urban (× 53.69)	86.8 (15.2)***	–2.5 (1.8)
Percent high school graduate (× 12.70)	13.8 (19.5)	–1.3 (2.4)
Percent unemployed (× 2.90)	6.1 (10.5)	3.3 (1.3)**
Percent black (× 10.04)	3.9 (6.4)	5.2 (0.8)***
Percent Hispanic (× 4.19)	0 (3.2)	0.4 (0.4)
Median age (× 4.60)	–110.3 (16.9)***	1.5 (2.1)
Percent > 65 years old (× 4.97)	158.5 (18.9)***	–0.3 (2.3)
Migration 1980s, 55- to 60-year-olds (× 12)	–86.3 (11.6)***	—
Migration 1980s, 60- to 64-year-olds (× 14)	275.9 (12.8)***	—
Migration 1980s, 70- to 74-year-olds (× 10)	—	–0.6 (0.8)
Migration 1980s, ≥ 75-year-olds (× 10)	—	9.4 (1.2)***
Migration 1990s, 65- to 70-year-olds (× 17)	–140.9 (13.5)***	—
Migration 1990s, 70- to 74-year-olds (× 12)	333.7 (11.6)***	—
Migration 1990s, ≥ 75-year-olds (× 13)	—	11.6 (0.9)***
Migration 2000s, ≥ 75-year-olds (× 15)	317.6 (7.1)***	2.9 (0.7)***
Regional indicators	Included	Included
*R*^2^	0.89	0.39
Number of counties	2,996^*b*^	2,996^*b*^
Abbreviations: IQR, interquartile range; $P_{85–94}^{\mathrm{EA}}$, (number of people 85–94 years old in 2010 divided by number of people 70–74 years old in 1980) × 10,000; $P_{100–104}^{\mathrm{EA}}$, (number of people 100–104 years old in 2010 divided by number of people 70–74 years old in 1980) × 10,000; PM_2.5_, particulate matter with aerodynamic diameter < 2.5 μm; SE, standard error. ^***a***^Estimates (standard error) of the difference in the rates of individuals 85–94-years old or 100–104 years old in 2010, over 10,000 individuals who were in the corresponding age group (55–64 years old or 70–74 years old) in 1980. The two parameters represent the probability of exceptional aging in 2010, given the corresponding starting population in 1980, and were labeled $P_{85–94}^{\mathrm{EA}}$ and $P_{100–104}^{\mathrm{EA}}$, respectively. Results shown were obtained from regression models fitting all the variables listed in the table. ^***b***^The models excluded all outliers, defined as observations with residuals that were > 3 standard deviations > 0 or < 0. Number of counties included in the analysis varied because of different numbers of outliers excluded from the data for 85- to 94-year-olds and for 100- to 104-year-olds. *, *p* < 0.05; **, *p* < 0.01; ***, *p* < 0.001.		

As shown in Table 2, the regression models estimated that each interquartile range (4.19 μg/m^3^) increase in PM_2.5_ was associated with 93.7 [standard error (SE) = 12.2] fewer remaining individuals in the 85- to 94-year-old group (*p* < 0.001) and 3.5 (SE = 1.5) fewer remaining individuals in the 100- to 104-year-old group (*p* < 0.05). Both estimates are scaled to a starting population of 10,000 individuals in 1980 in the corresponding age groups and represent the difference in the probability of reaching 85–94 years of age or 100–104 years of age in 2010 associated with PM_2.5_ levels.

### Socioeconomic Determinants and Probabilities of Exceptional Aging

Other determinants also had significant negative associations with the probability of exceptional aging (Table 2). The cigarette smoking rate was the strongest determinant of exceptional aging, with an interquartile range (4.77%) increase in smoking rate associated with 181.9 (SE = 14.0) and 6.4 (SE = 1.7) fewer remaining individuals in the 85- to 94-year-old and the 100- to 104-year-old groups, respectively. Differences across counties in obesity rates and median income were associated with exceptional aging for both age groups (Table 2). As previously reported (Masters 2012), counties with higher percentages of black residents had greater aging to 100–104 years. Aging to 85–94 years showed strong associations with median age and percentage of population > 65 years old. For centenarians, there were no associations with median age or percent > 65 years old. For both age groups, there were very strong associations with relevant age-specific migration rates, which were generally consistent with expected structural associations (Table 2).

### Sensitivity Analysis and Possible Influence of Migration Rates

**Figure 2:**
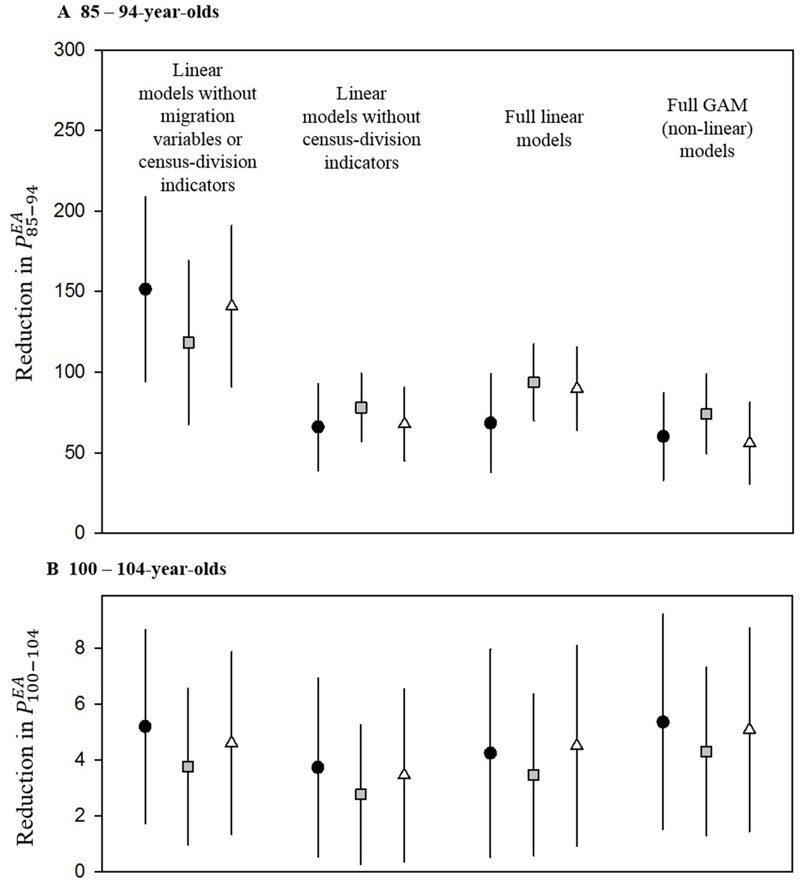
Estimated reduction in indices of exceptional aging, *P^EA^*_85–94_ (*A*), and *P^EA^*_100–104_ (*B*), associated with an interquartile range increase in PM_2.5_ (4.19 μg/m^3^) for various models. Black circles represent models with no censored observations, gray squares represent models excluding observations with residuals > 3 standard deviations from zero, and open triangles represent models excluding observations with the 5% most extreme migration patterns based on the migration rate for ≥ 75-year-olds in 2000. *P^EA^*_85–94_, (number of people 85–94 years old in 2010 divided by number of people 70–74 years old in 1980) × 10,000; *P^EA^*_100–104_, (number of people 100–104 years old in 2010 divided by number of people 70–74 years old in 1980) × 10,000; PM_2.5_, particulate matter with aerodynamic diameter < 2.5 μm.

As illustrated in Figure 2, the associations of PM_2.5_ with exceptional aging were robust and stable in sensitivity analyses using alternative regression models. Controlling for migration variables attenuated the PM_2.5_ effect somewhat for the 85- to 94-year-old group but not for the 100- to 104-year-old group. Models with and without regional indicator variables and GAMs that allowed for non-linear associations with all other covariates resulted in remarkably similar estimates. Furthermore, using different strategies to censor for extreme outliers to evaluate the potential influence of outliers provided remarkably consistent statistically significant results in all cases. The largest county in the analysis, Los Angeles, was the most heavily weighted and was one of the most highly polluted counties (Figure 1). Los Angeles, however, tended to have more exceptional aging than was predicted by the model; therefore, its inclusion slightly mitigated the negative linear associations between PM_2.5_ and exceptional aging.

To further evaluate the possible influence of migration rates on our results, we examined whether migration was differential for PM_2.5_ concentrations, that is, whether individuals moved from communities with high PM_2.5_ into communities with low PM_2.5_, or vice versa. We found minimal or no correlation between PM_2.5_ and the relevant migration rates, including the migration rate for 55- to 60-year-olds in 1980s (*r* = –0.03), the migration rate for 60- to 64-year-olds in the 1980s (*r* = –0.07), the migration rate for 70- to 74-year-olds in the 1980s (*r* = –0.07), the migration rate for ≥ 75-year-olds in the 1980s (*r* = –0.08), the migration rate for 65- to 70-year-olds in the 1990s (*r* = –0.07), the migration rate for 70- to 74-year-olds in the 1990s (*r* = 0.01), the migration rate for ≥ 75-year-olds in 1990s (*r* = 0.14), and the migration rate for ≥ 75-year-olds in the 2000s (*r* = 0.21).

### Linearity of the Association of PM_2.5_ with Probabilities of Exceptional Aging

**Figure 3:**
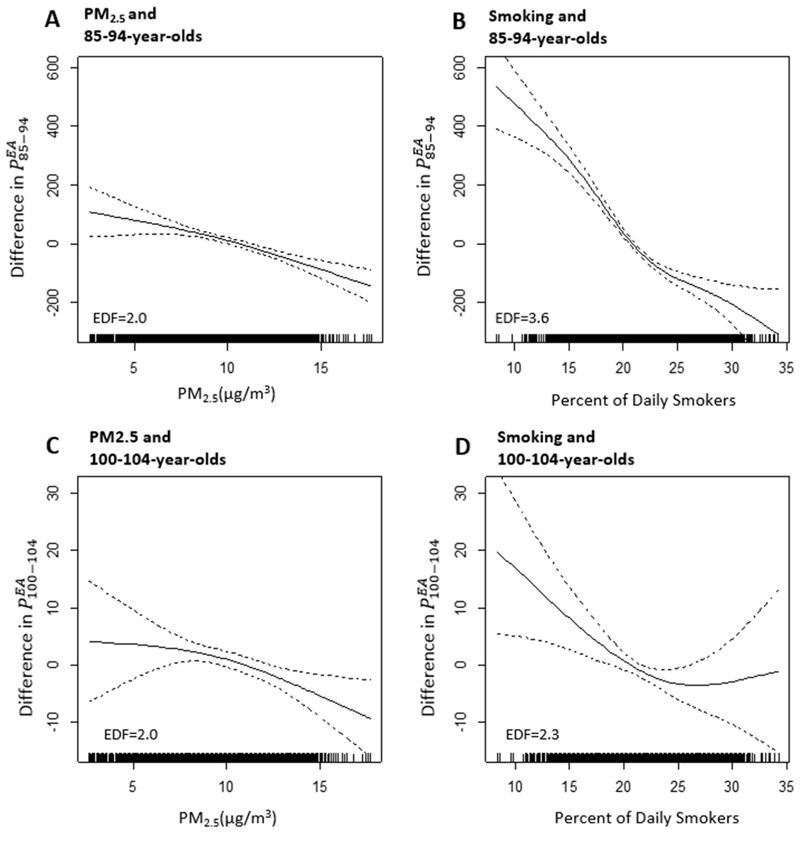
Nonparametric smoothed functions illustrating relationships between the indices of exceptional aging (*P^EA^*_85–94_ and *P^EA^*_100–104_) and PM_2.5_ (*A*,*C*) or percentage of daily smokers (*B*,*D*). Equivalent degrees of freedom (EDFs) are reported in each panel. *P^EA^*_85–94_, (number of people 85–94 years old in 2010 divided by number of people 70–74 years old in 1980) × 10,000; *P^EA^*_100–104_, (number of people 100–104 years old in 2010 divided by number of people 70–74 years old in 1980) × 10,000; PM_2.5_, particulate matter with aerodynamic diameter < 2.5 μm.

The GAMs that allowed for nonlinear associations with PM_2.5_ and all other covariates (except for the census-division indicators) indicated that the association of PM_2.5_ levels with the probability of exceptional aging for both age groups (*P^^EA^^*_85–94_ and *P^^EA^^*_100–104_) was approximately linear. Figure 3 presents plots of nonparametric smoothed functions (Figure 3A,C). For comparison, plots of the relationship between exceptional aging and percentage of smokers are also included (Figure 3B,D). For PM_2.5_, the penalized regression smoother was not significantly different from linear; however, for illustrative purposes, Figure 3 presents the estimated smooth with 2 degrees of freedom. In addition, as shown in Figure 2, GAMs with a linear fit for PM_2.5_ but spline smooth functions for the other covariates (except for the census-division indicators) resulted in linear associations for PM_2.5_ that were nearly the same as those from the full linear models.

## Discussion

In this nationwide analysis of ~28 million individuals ≥ 55 years old across 3,034 counties, higher levels of PM_2.5_ air pollution were associated with lower population-based probabilities of exceptional aging, even after adjusting for smoking, obesity, demographic and socioeconomic variables, total and age-specific migration rates, and differences across the nine census divisions of the United States. The highest rates of exceptional aging occurred in counties with relatively low pollution levels, lower rates of smoking and obesity, and higher median income.

Prospective cohort survival studies that follow individuals over time can control for many individual risk factors and have provided some of the most important evidence regarding the health effects of long-term exposures to air pollution (Beelen et al. 2014; Cesaroni et al. 2013; Crouse et al. 2012; Dockery et al. 1993; Jerrett et al. 2013; Miller et al. 2007; Pope et al. 2002; Zeger et al. 2008). The ecological approach used in the present analysis provides alternative evidence that is a simple, direct, and transparent exploration that used U.S. county-level census and related data that are easily accessible and publicly available. Our approach did not require probabilistic data linkage with death records, nor was there a need to specify cause of death. This approach may overcome limitations regarding health assessment of the effects of air pollution, as well as other health determinants, in countries where population data are available, but the quality and/or availability of mortality data is limited. Furthermore, if regular and reliable population counts are available, repeated analysis could be used to evaluate time trends in risk.

The weighted regression techniques used in our analysis are also relatively straightforward and easy to interpret. For example, the results of this analysis demonstrate the relatively large effect of smoking rates. On average, out of 10,000 persons who were 55–65 years old in 1980, ~2,295 persons survived to be 85–94 years old in 2010. The regression coefficients on smoking suggest that, for every 1% increase in smoking rate, there were approximately 38 fewer 85- to 94-year-olds alive in 2010. These results suggest that the current average smoking rate of 18% in the United States (Agaku 2014) is responsible for a reduction of the probability of reaching age 85–94 years by ~30% {[(38 × 18) ÷ 2,295] × 100}. By comparison, regression results suggest that a 10-μg/m^3^ increase in long-term exposure to PM_2.5_ is associated with ~225 fewer 85- to 94-year olds or a reduction in the probability of reaching age 85–94 years by ~9.7% [(223 ÷ 2,295) × 100]. Comparisons between the effects of smoking and air pollution are similar for the probability of reaching 100–104 years of age.

We also compared the effects of various other factors and estimated reductions in exceptional aging probabilities associated with interquartile-range changes in each population-based variable. Based on effect estimates presented in Table 2, if we could improve any of these factors, the largest increase in exceptional aging would come from reduced smoking. Reductions in obesity, poverty, and air pollution would also provide substantial improvements. Other unmeasured differences resulting in racial inequalities or in residual uncontrolled confounding may contribute to these associations.

The most important limitation to our approach concerns population mobility. The constructed indices of exceptional aging would be ideal if there were no migration across counties, and the populations in these counties could therefore be treated like cohorts that were being followed over time. However, because these populations are not strictly cohorts but are populations with uncontrolled migration, we controlled for migration as part of the regression analysis by including in the regression models age-specific migration rates that provided the closest possible temporal alignment consistent with the initial age group and with relevant age groups in subsequent decades, and the migration data available for all three decades. Migration rates did not appear to operate as serious confounders, as indicated by the similar associations of PM_2.5_ with exceptional aging found in unadjusted data (Figure 1), covariate-adjusted models (Table 2 and Figure 2), and the weak correlations between PM_2.5_ and the age-specific migration rates. Because persons move to and from counties with different levels of pollution, however, even full population–based adjustments could not fully account for resulting misclassifications of PM_2.5_ exposure.

Estimated pollution levels were limited to the years 1999–2008 in our analysis because PM_2.5_ data were not collected regularly in the United States until 1999. Previous analyses have shown robust spatial patterns in the ranking order of PM_2.5_ levels measured at different U.S. locations between 1980 and 1999 (Pope et al. 2002). Therefore, the 1999–2008 subperiod can be considered a suitable proxy of levels across the entire 1980–2010 period. There is also some temporal mismatch between the three-decade time period used for aging (1980–2010) and the estimates for obesity prevalence (2004–2010) and smoking and socioeconomic variables (approximately 2000). The association estimates may be not be significantly affected if the spatial contrasts of these variables are reasonably stable over time.

The analysis was conducted at a population, not an individual, level; therefore, this approach cannot evaluate risk factors at the individual level or explore sensitive subpopulations. Other unmeasured factors might also influence exceptional aging and result in residual confounding. Finally, information on age was obtained from U.S. census data and was not independently validated. This limitation may be particularly relevant for the 100- to 104-year-old group because birth records were less accurate over a century ago (Sachdev et al. 2012) than in more recent decades. Nevertheless, results were mostly similar for 85- to 94-year-olds and 100- to 104-year-olds. Additionally, age errors are likely to be nondifferential with respect to the exposures and to bias our analysis toward the null rather than generate the observed significant associations.

The present analysis is population-based, has comprehensive coverage of the United States, and includes a large number of exceptionally aged individuals. In particular, because of the large population of the United States and its relatively high life expectancy, the 48,303 centenarians in our data represent approximately 11% of all centenarians worldwide (United Nations 2010). We excluded potential influences from outliers, including counties with large in- or out-migration rates for elderly individuals. Therefore, our analysis provides statistically robust results that might apply to other countries with similar age structures and possibly to other regions in which life expectancy is increasing.

## Conclusions

Our study supports the association between long-term exposure to air pollution and probability of exceptional aging. This association was found in our analysis—at least for part of the PM_2.5_ distribution—even at PM_2.5_ concentrations below the annual average limit values set by the U.S. Environmental Protection Agency (2013) (12 μg/m^3^), as well those set by other countries, such as Japan (15 μg/m^3^; Ministry of the Environment 2009), the European Union (25 μg/m^3^; European Commission 2008), and China (15–35 μg/m^3^; Chinese Ministry of Environmental Protection). Rates of smoking, obesity, and poverty also showed associations with exceptional aging. Although more studies in other nations are needed, particulate matter air pollution is ubiquitous, and on the basis of our results, reducing PM_2.5_—along with improving other sources of inequality—may contribute to increasing the probability of exceptional aging.

## Supplemental Material

(385 KB) PDF
