# Supplementary material for: Particulate Air Pollution, Exceptional Aging, and Rates of Centenarians: A Nationwide Analysis of the United States, 1980–2010
Source: Environ Health Perspect. 2016 May 3;124(11):1744–50. doi: 10.1289/EHP197 (PMC5089884; doi:10.1289/EHP197)
Supplement: (385 KB) PDF [file EHP197.s001.acco.pdf]

Environ Health Perspect
DOI: 10.1289/EHP197

Note to readers with disabilities: *EHP* strives to ensure that all journal content is accessible to all readers. However, some figures and Supplemental Material published in *EHP* articles may not conform to 508 standards due to the complexity of the information being presented. If you need assistance accessing journal content, please contact ehp508@niehs.nih.gov. Our staff will work with you to assess and meet your accessibility needs within 3 working days.

Supplemental Material

Particulate Air Pollution, Exceptional Aging, and Rates of Centenarians: A Nationwide Analysis of the United States, 1980–2010

Andrea A. Baccarelli, Nick Hales, Richard T. Burnett, Michael Jerrett, Carter Mix, Douglas W. Dockery, and C. Arden Pope III

Table of Contents

Table S1. Results of different regression models, including estimates (standard errors) of difference in probability of aging to 85-94 years (P_{85-94}^{EA}) associated with 10 μg/m^3 of $PM_{2.5}$, adjusted for smoking, migration, demographic, socioeconomic, and regional indicator variables. The results reported in the paper are from the 'primary model-censored' model (shaded in grey).

Table S2. Results of different regression models, including estimates (standard errors) of difference in probability of aging to 100-104 years ($P_{100-104}^{EA}$) associated with 10 μg/m^3 of $PM_{2.5}$, adjusted for smoking, migration, demographic, socioeconomic, and regional indicator variables. The results reported in the paper are from the 'primary model-censored' model (shaded in grey).

Table S1. Results of different regression models, including estimates (standard errors) of difference in probability of aging to 85-94 years (P^{EA}_{85-94}) associated with 10 μg/m^3 of $PM_{2.5}$, adjusted for smoking, migration, demographic, socioeconomic, and regional indicator variables. The results reported in the paper are from the 'primary model-censored' model (shaded in grey).

	**No region adjustment**	**No region adjustment-censored†**	**Primary model**	**Primary model-censored†**	**Robust regression‡**	**Robust regression‡-censored†**
$PM_{2.5}$ (x10 μg/m^3)	-171.7 (36.4)***	-149.6 (31.2)***	-267.3 (40.9)***	-225.2 (35.0)***	-175.1 (21.4)***	-174.8 (21.5)***
% Smoking	-46.8 (3.9)***	-49.6 (3.4)***	-37.4 (4.1)***	-39.4 (3.5)***	-39.0 (2.7)***	-38.9 (2.7)***
% Obesity	-8.1 (3.4)*	-5.7 (2.9)	-20.4 (3.8)***	-16.8 (3.2)***	-12.9 (2.3)***	-12.7 (2.3)***
Population density	4.2 (2.0)*	4.0 (1.7)*	6.4 (2.0)**	5.9 (1.7)**	6.2 (0.8)***	6.3 (0.8)***
% Urban	2.7 (0.4)***	2.3 (0.3)***	2.5 (0.4)***	2.1 (0.3)***	1.3 (0.3)***	1.3 (0.3)***
% Below poverty	-16.1 (3.9)***	-21.1 (3.4)***	-13.0 (4.0)**	-17.6 (3.4)***	-19.5 (2.9)***	-19.8 (2.9)***
Median income	5.9 (1.8)**	1.9 (1.6)	7.5 (1.8)***	3.5 (1.6)*	-2.8 (1.0)**	-2.8 (1.0)**
% High school graduate	0.8 (2.1)	1.0 (1.8)	3.5 (2.1)	3.5 (1.8)	4.0 (1.5)**	4.1 (1.5)**
% Unemployed	-0.5 (4.6)	0.9 (4.0)	4.8 (5.1)	3.6 (4.3)	-1.0 (4.0)	-1.2 (4.0)
% Black	-4.0 (0.8)***	-4.3 (0.7)***	-3.8 (0.9)***	-3.7 (0.7)***	-4.2 (0.6)***	-4.1 (0.6)***
% Hispanic	-4.5 (1.0)***	-3.4 (0.9)**	-3.9 (1.0)**	-2.8 (0.9)**	-2.5 (0.7)**	-2.4 (0.7)**
Median age	-47.0 (5.0)***	-38.1 (4.4)***	-50.8 (5.2)***	-41.7 (4.5)***	-32.1 (3.8)***	-32.2 (3.8)***
% Over 65 years old	56.7 (5.0)***	45.8 (4.4)***	58.1 (5.3)***	47.4 (4.6)***	28.2 (4.1)***	28.1 (4.1)***

Continued on the following page

Table S1. Continued from the previous page

	**No region adjustment**	**No region adjustment-censored†**	**Primary model**	**Primary model-censored†**	**Robust regression‡**	**Robust regression‡-censored†**
% Migration	34.1 (3.2)***	33.6 (2.8)***	30.7 (3.2)***	31.0 (2.8)***	29.6 (2.5)***	28.8 (2.5)***
Migration rate, 60–64-year-olds	-1.4 (1.2)	-3.5 (1.0)**	-1.2 (1.2)	-3.3 (1.0)**	-3.5 (1.1)**	-3.4 (1.1)**
Migration rate, 65–69-year-olds	8.6 (1.5)***	9.4 (1.3)***	8.1 (1.5)***	9.1 (1.3)***	6.4 (1.5)***	6.5 (1.5)***
Migration rate, 70–74-year-olds	18.9 (1.4)***	19.0 (1.3)***	18.6 (1.4)***	18.6 (1.2)***	17.2 (1.3)***	17.3 (1.3)***
Migration rate, 75–79-year-olds	19.6 (0.8)***	18.1 (0.7)***	20.2 (0.8)***	18.7 (0.7)***	18.3 (0.6)***	18.2 (0.6)***
Regional indicators	No	No	Yes	Yes	Yes	Yes
R^2	0.82	0.83	0.83	0.84	0.58‡	0.59‡
No. of counties	3034	2987	3034	2987	3034	2987

*p<0.05; **p<0.01; ***p<0.001

†Censored models excluded all outliers, defined as observations with residuals that were more than three standard deviations greater than or less than zero.

‡Robust regression. Note that for robust regression models, robust version[23] of R^2 is presented. R^2 values obtained from robust regression models are therefore not directly comparable to those from other models.

Table S2. Results of different regression models, including estimates (standard errors) of difference in probability of aging to 100-104 years ($P^{EA}_{100-104}$) associated with 10 μg/m^3 of $PM_{2.5}$, adjusted for smoking, migration, demographic, socioeconomic, and regional indicator variables. The results reported in the paper are from the 'primary model-censored' model. (shaded in grey).

	**No region adjustment**	**No region adjustment-censored†**	**Primary model**	**Primary model-censored†**	**Robust regression‡**	**Robust regression‡-censored†**
$PM_{2.5}$ (x10 μg/m^3)	-9.9 (4.0)*	-8.3 (3.1)**	-11.9 (4.6)**	-10.4 (3.6)**	-11.7 (2.4)***	-11.7 (2.4)***
% Smoking	-2.0 (0.4)***	-1.8 (0.3)***	-1.7 (0.5)**	-1.4 (0.3)***	-1.1 (0.3)**	-1.1 (0.3)**
% Obesity	-0.6 (0.4)	-0.2 (0.3)	-1.2 (0.4)**	-0.8 (0.3)**	-1.0 (0.3)***	-1.0 (0.3)***
Population density	0.4 (0.2)	0.4 (0.2)*	0.4 (0.2)	0.4 (0.2)*	0.5 (0.1)***	0.5 (0.1)***
% Urban	0.0 (0.0)	0.0 (0.0)	0.0 (0.0)	0.0 (0.0)	0.0 (0.0)	0.0 (0.0)
% Below poverty	-0.3 (0.4)	-0.6 (0.3)	0.1 (0.4)	-0.3 (0.4)	-0.2 (0.3)	-0.2 (0.3)
Median income	0.5 (0.2)**	0.5 (0.2)**	0.7 (0.2)**	0.5 (0.2)**	0.5 (0.1)***	0.5 (0.1)***
% High school graduates	-0.1 (0.2)	0.0 (0.2)	-0.1 (0.2)	0.0 (0.2)	0.0 (0.2)	0.0 (0.2)
% Unemployed	0.5 (0.5)	0.5 (0.4)	1.0 (0.6)	0.8 (0.4)	0.3 (0.4)	0.3 (0.4)
% Black	0.4 (0.1)***	0.4 (0.1)***	0.3 (0.1)**	0.4 (0.1)***	0.4 (0.1)***	0.4 (0.06)***
% Hispanic	-0.1 (0.1)	0.1 (0.1)	-0.1 (0.1)	0.1 (0.1)	0.1 (0.1)	0.1 (0.1)
Median age	-0.6 (0.6)	-1.1 (0.4)*	-0.3 (0.6)	-0.9 (0.5)	-1.7 (0.4)***	-1.7 (0.4)***
% Over 65	1.0 (0.6)	1.7 (0.4)**	0.6 (0.6)	1.3 (0.5)**	2.0 (0.4)***	2.0 (0.4)***

Continued on the following page

Table S2. Continued from the previous page

	**No region adjustment**	**No region adjustment-censored†**	**Primary model**	**Primary model-censored†**	**Robust regression‡**	**Robust regression‡-censored†**
% Migration	0.2 (0.4)	0.4 (0.3)	0.1 (0.4)	0.3 (0.3)	0.6 (0.3)*	0.6 (0.3)*
Migration rate, 60–64-year-olds	0.2 (0.1)	0.2 (0.1)	0.2 (0.1)	0.2 (0.1)	0.2 (0.1)	0.2 (0.1)
Migration rate, 65–69-year-olds	-0.2 (0.2)	-0.2 (0.1)	-0.2 (0.2)	-0.1 (0.1)	-0.1 (0.2)	-0.1 (0.2)
Migration rate, 70–74-year-olds	0.1 (0.2)	0.0 (0.1)	0.2 (0.2)	0.0 (0.1)	-0.2 (0.1)	-0.2 (0.1)
Migration rate, 75–79-year-olds	1.3 (0.1)***	1.2 (0.1)***	1.3 (0.1)***	1.3 (0.1)***	1.4 (0.1)***	1.3 (0.1)***
Regional indicators	No	No	Yes	Yes	Yes	Yes
R^2	0.29	0.35	0.3	0.37	0.3‡	0.31‡
No. of counties	3032	2997	3032	2997	3032	2997

*p<0.05; **p<0.01; ***p<0.001

†Censored models excluded all outliers, defined as observations with residuals that were more than three standard deviations greater than or less than zero.

‡Robust regression. Note that for robust regression models, robust version[23] of R^2 is presented. R^2 values obtained from robust regression models are therefore not directly comparable to those from other models.
